# The Co-Piloting Model for Using Artificial Intelligence Systems in Medicine: Implementing the Constrained-Disorder-Principle-Based Second-Generation System

**DOI:** 10.3390/bioengineering11111111

**Published:** 2024-11-03

**Authors:** Yaron Ilan

**Affiliations:** Department of Medicine, Hadassah Medical Center, Faculty of Medicine, Hebrew University, Jerusalem 9112001, Israel; ilan@hadassah.org.il

**Keywords:** digital health, artificial intelligence, robots, medical care, future medicine

## Abstract

The development of artificial intelligence (AI) and machine learning (ML)-based systems in medicine is growing, and these systems are being used for disease diagnosis, drug development, and treatment personalization. Some of these systems are designed to perform activities that demand human cognitive function. However, use of these systems in routine care by patients and caregivers lags behind expectations. This paper reviews several challenges that healthcare systems face and the obstacles of integrating digital systems into routine care. This paper focuses on integrating digital systems with human physicians. It describes second-generation AI systems designed to move closer to biology and reduce complexity, augmenting but not replacing physicians to improve patient outcomes. The constrained disorder principle (CDP) defines complex biological systems by their degree of regulated variability. This paper describes the CDP-based second-generation AI platform, which is the basis for the Digital Pill that is humanizing AI by moving closer to human biology via using the inherent variability of biological systems for improving outcomes. This system augments physicians, assisting them in decision-making to improve patients’ responses and adherence but not replacing healthcare providers. It restores the efficacy of chronic drugs and improves adherence while generating data-driven therapeutic regimens. While AI can substitute for many medical activities, it is unlikely to replace human physicians. Human doctors will continue serving patients with capabilities augmented by AI. The described co-piloting model better reflects biological pathways and provides assistance to physicians for better care.

## 1. Introduction

Computer-controlled machines are generated to perform activities that demand human cognitive function. There is a growing improvement in artificial intelligence (AI) and machine learning (ML)-based digital health systems for use in healthcare. The potential use of AI in healthcare includes disease diagnosis, drug development, treatment personalization, and gene editing [1]. It has led companies and physicians to consider the concept that, at some point, physicians will be replaced by robots. Vindo Koshla, the legendary Silicon Valley investor, argued that machines would replace 80 percent of doctors in the future of healthcare [2]. It was suggested that machines could replace doctors because professional work can be fragmented into routine and process-based parts requiring judgment, creativity, or empathy [3]. Technological singularity (TS) implies a time when AI surpasses human intelligence and involves using AI to take full autonomy and responsibility for decision-making. TS in healthcare suggests replacing medical practitioners with AI-enabled robots and peripheral systems [4].

However, patients and caregivers using AI-based systems in routine care must catch up to expectations.

This paper reviews several challenges healthcare systems face and the obstacles of integrating digital systems into routine patient care. The constrained disorder principle (CDP) defines complex biological systems by their inherent variability [5]. The paper focuses on integrating digital systems with human physicians. It describes the CDP-based second-generation AI systems designed to move closer to biology and reduce complexity, aiding but not replacing physicians to improve patient outcomes [6].

## 2. Currently Used AI Systems in Healthcare

Machine learning (ML) and natural language processing (NLP) are two common areas of AI. In ML, the system gathers and examines structured data such as gene traits and diagnostic images. NLP involves collecting unstructured data from electronic medical records (EMRs) [7]. Both can assist in clinical decisions. ML systems improve algorithms by automatically using data and cognitive inputs without being programmed for them [8]. It learns patterns used for prediction from large amounts of information [8,9].

Knowledge-based systems are designed to simulate human information by requesting experts to provide the methods they use to solve problems [10]. Data from wearable and non-wearable biosensors, physical sensors, and EMR provide sources for big data, which serve as tools to improve the accuracy and efficiency of diagnosis and treatment [8,11,12,13]. Precision medicine-based AI systems consider individual subjects and their genomic variations, contributing factors, age, gender, ethnicity, and emotional state, and tailor interventions accordingly [12,14].

AI-based systems can gather and analyze large amounts of data and draw conclusions, which is challenging for human physicians [13]. They can help predict disease, prepare long-term plans for patient care, improve care accuracy, reduce side effects, and personalize treatments 15. AI systems use algorithms for data processing using several methods, including artificial neural networks (ANNs), fuzzy expert systems (FESs), evolutionary calculations (ECs), and hybrid intelligent systems (HISs) [4].

Some theories suggest that ANN mimics human thinking through data collection, analysis of examples of answers to previous problems, and the use of learning algorithms, based on which a response is designed [15]. The type of data being processed governs the value of the results. A deep neural network (DNN) is an ANN that comprises more layers, enabling better predictions from data [16]. It benefits from the training dataset size. Deep learning (DL) recognizes complex correlations to be extracted by ML algorithms. Compared with earlier networks that use three to five connections, DL uses ANN with more than ten layers, encompassing an architecture within layers and computational actions like tensors. The FES uses professional information in a particular area that mimics an expert’s response. EC is a method based on a biological evaluation that provides a solution. HIS is a synergetic model that is based on some of these systems [17,18,19].

AI and robots are used in numerous areas to replace the human workforce and assist human performance [20]. Systems are divided into assistive and autonomous. Assistive systems improve performance through data collection and analysis, leading to the conduction of tasks under human supervision [21]. Examples are robots integrated into prosthetics and implants, robotic assistants for patient assistance and rehabilitation, and robots that assist healthcare staff with their jobs [22,23]. Autonomous systems are designed to react to real-world situations; they function with minimal human interaction and make decisions. Examples are digital receptionists and autonomous implanted devices [24].

Speech recognition and other interface systems can regulate robotics. Examples are simulations of social interaction, cognitive stimulation, and health assessment [25,26]. AI platforms collect and analyze data for object identification, language processing, facial recognition, and improving diagnosis [27,28]. In surgery, robotics can undertake pre-programmed tasks under a surgeon’s control. Surgeons can integrate pre-op data with real-time operating parameters for improving outcomes [29,30]. Semi-active systems enable surgeons to supplement the pre-programmed component and use master–slave systems that lack autonomous elements and depend entirely on their actions [31].

AI-based monitoring approaches support healthcare providers in diagnosing symptoms and providing prognosis [28,32]. ML can detect abnormality with contextual factors, such as an object, time, space, and duration for decision support on the suitable action [33]. It predicts the risk of postoperative complications with better accuracy than conventional approaches [34]. ML can improve cardiovascular risk prediction by correlating complex interactions between risk factors [35].

Computer vision (CV) involves image processing, pattern recognition, and response [28,36]. It can be used for radiology and pathology for image processing [4]. Supervised cancer detection implies that the machine has “learned” mapping from a tagged dataset; unsupervised learning is essentially clustering, regardless of the task. Supervised learning can be used to predict diagnosis and prognosis in sepsis when fed large datasets; reinforcement training is a hybrid that combines the other two [37,38]. AI algorithm-based grading systems can assess the eye fundus photographs of diabetic patients [39].

While these systems offer numerous benefits, they pose challenges for end users and healthcare systems.

## 3. The Challenges Faced by Current AI Systems: Low Engagement by Patients and Physicians

The digital health market has ballooned to 350,000 products: apps, wearables, and telemedicine devices [40,41]. The market reached USD 116.39 billion in 2019 and is expected to exceed USD 833.44 billion by 2027 [42]. The market size of AI healthcare is likely to grow by 40% compound annually [20]. AI-based systems are predicted to reduce annual US healthcare costs by USD 150 billion in 2026. Savings are expected by changing the healthcare model from reactive to proactive, centered on health management rather than treatments. It is expected to reduce doctor visits and hospitalizations [20]. While the AI field is increasingly growing, multiple challenges prevent it from expanding. Patients, physicians, and healthcare authorities have a low level of engagement [43,44].

Healthcare systems are becoming proactive following the P5 concept, which represents personalized, predictive, participatory, preventive, and precision [45]. This patient-centered model sets the basis for AI platforms that assist in diagnosis and treatment decision-making [8]. However, these systems are associated with numerous challenges, such as privacy, ethical principles, safety, security, and various biases, which may result in harmful decisions [8].

There is a tendency among healthcare professionals to refrain from resisting or ignoring new technologies. There are numerous reasons for the lack of engagement, such as the perceived threats to professional status, privacy and autonomy concerns, and ethical and legal issues of responsibility [46,47,48]. The low engagement with digital systems led to the “valley of death of AI” or “AI winter” [8]. Machines are expected to perform repetitive, routine tasks under foreseeable environments and collaborate with subjects to conduct activities in uncontrolled situations [49,50,51]. However, there are no validation data for the functions machines can perform. It is thus not always clear how a robot can assist healthcare providers and how forecasts of outcomes can change managed care [52,53].

The quality and reliability of digital health devices and sensor inputs still need to be determined [54]. Algorithms still underperform in unusual cases of treatment resistance and side effects and where there are no previous examples to build on [55]. Digital mammography has a sensitivity of 84% for detecting breast cancers and is yet to be improved [56,57].

AI technologies achieved notable results in predicting sepsis or cardiovascular risk and monitoring vital parameters in intensive care units and robots. However, their use in real-world settings is still limited [8]. Outcomes drive precision patient management and aim for optimal outcome prediction, but this is challenging to achieve using the current AI-based methods [58]. Computers can search more datasets faster than humans, but speed and efficiency do not always translate to accuracy [59]. Humans still perform many tasks and play an essential role in determining AI-based systems’ course of operation [60]. A few AI tools achieved regulatory clearance due to their performance in limited studies [61].

Explainability is an essential tool for justifying AI-based decisions. It can help validate predictions, improve models, and provide new insights into the problem, leading to more reliable AI systems [62]. Explainability is a feature that enables a reconstruction of why an AI system produces a certain decision [63]. AI-based predictions are not always explainable, in contrast to rule-based systems. This may lead to hiding errors or biases. The lack of explainability makes it hard for physicians to judge the reliability of the AI output [60]. Explainable AI involves the strategies and methodologies used to construct AI systems that enable end users to understand and interpret the outputs and predictions made by AI models [62]. The increasing use of opaque AI applications in high-stakes fields, especially healthcare, has increased the need for clarity and explainability. This is due to the potentially high-impact consequences of incorrect AI predictions in such critical sectors. The effective integration of AI models in healthcare depends on these models’ ability to be both explainable and interpretable. Gaining the trust of healthcare professionals requires AI applications to be transparent about their decision-making processes and underlying logic [64]. The challenges of accountability, responsibility, and liability for harm caused by AI are unresolved. It is unclear who is responsible: the programmer, manufacturer, end user, AI/robotic system, or dataset provider. The European Parliament’s Resolution assigned the responsibility to the human factor, including the developer, manufacturer, owner, or operator [65].

The P5 model for healthcare is used to develop individualized care and is associated with multiple factors: socio-economic background, gender, ethnicity, and education. However, it has numerous potential biases toward particular groups of the population. Training datasets face the challenge of under-representing subgroups, and essential variables are dispersed differently across subgroups. Examples are cardiovascular disease and Parkinson’s disease, which have different progression based on gender. This results in unintended and undesirable discrimination bias [66,67]. Implementations of AI can account for ethnicity, gender, and other differences, generating more personalized results and a “desirable bias” [68].

The costs associated with using newer versions of AI systems are expected to be economic burdens on health services. They also require higher computing power, which is not always available [8].

Among the many challenges healthcare systems face is low adherence to care, where 40% of patients need to adhere to their regimens [69]. Many patients go through more than one line of treatment to reach an optimal response and cannot achieve it. A third to half of the patients with chronic diseases suffer from a partial or complete loss of effect of chronic therapies, a significant cause of loss of adherence to chronic therapies [70]. Low adherence and loss of response in patients with chronic diseases are substantial burdens on health systems, leading to increased admissions and the need for expensive drugs, which are not always better.

The lack of patients’ adherence to AI-based apps is a significant challenge [71]. Low engagement of patients and physicians with the currently used digital health apps prohibits their use as a solution for the loss of efficacy and low adherence to chronic therapies [60,72,73]. Digital health apps are subject to significantly reduced engagement rates, although their availability is increasing. There needs to be more sustainability of use, and many users must adhere to these apps as intended. Studies suggest that 80 percent of subjects exposed to digital health interventions only engage at a minimum level. They only log into the app once and do not use it consistently long-term [74]. It is estimated that less than 5% of patients show long-term adherence to apps [75,76]. The ethical dilemmas that occur between AI and patients are also challenged [77]. A recent study based on multiple interviews documented marked variability in patients’ perspectives on AI systems; there was no single pattern regarding patients’ opinions on the introduction of robots or the functionalities and tasks that robots can perform [71]. Patients accept and value care automation while looking for reciprocity in robotics [78].

These encounters raise the challenges of whether robots can replace medical care providers.

## 4. Why Will Robots Not Replace Physicians?

Multiple studies thoroughly analyzed the challenge of replacing caregivers with AI-based systems. AI is expected to enhance human clinicians’ efficiency, improve diagnostic and treatment accuracy, and reduce costs while eliminating emotional contact in the patient–doctor relationship [79,80]. Empathy is an ability to understand and share feelings, which develops from self-awareness and interactions with other members of society [81]. Empathy cannot be replaced and is a challenge for AI-based interactions, which Artificial Empathy (AE) attempts to resolve. AI-enabled, empathetic mobile app technologies showed some promise in dealing with this challenge [82,83].

Although AI and robotics are performing well, human surveillance is still essential [60]. Human physicians have a non-linear working method that adapts to ever-changing conditions and quickly evolving situations, which is difficult to teach to a computer [20,59]. This is a significant challenge for ML-based technologies. Following pre-defined guidelines, diagnosis, and treatment require creativity and problem-solving skills that algorithms can never have. Patients vary, and no case is the same. This implies a need for a human physician to decide whether to use a data-derived means to diagnose or decide on a therapeutic regimen [84].

The human brain is complex and can oversee a vast data scale, so it is not worth developing an AI that attempts to imitate it, as the human brain does it well. This implies that while AI-based systems can assist and guide physicians, they may need to leave the complex analysis and decisions to humans 85. There are tasks that algorithms and robots cannot complete with humans in. A human physician must constantly review the data analysis conducted and the conclusions made by the AI system [85].

There will always be tasks where humans are faster, more reliable, and cheaper. Human physicians rely on more than just data for medical decision-making. Computer scientists find that physicians’ “gut feelings” that cannot be translated into algorithms influence decisions [86,87]. This implies that collaboration between humans and technology should be the ultimate goal.

## 5. Integrating the Human Brain with a Computer: The Future Physician

The practice of medicine is changing and is expected to change with improvements in AI methods [13]. Although technology has indeed come a long way, the focus on advancing technology should be on augmenting a physician’s workflow rather than replacing it. Human–machine collaborations are performing better than either one alone [61,86,87]. The role of AI in medicine is not about replacing physicians; instead, it is about optimizing and improving what they already achieve [13,88].

Intelligent digital technologies require competent professionals [89]. It is not tech vs. human, as complex technologies require skilled professionals. It is worthwhile for a program to assist in repetitive, data-based tasks. Internists spend a large proportion of their working hours on paperwork that can be automated [90]. Decision-making, on the other hand, needs to be supervised by a human physician. The analysis of a large amount of data for improving diagnostic approaches is limited by humans’ cognitive and sensory abilities, which are expected to be enhanced by AI [28].

AI can improve patient outcomes by 30% to 40% while reducing treatment costs by up to 50% [13,91]. It can improve efficiency and decrease costs by shifting human labor to more complex tasks, identifying workflow optimization strategies, reducing medical waste via better coordination, eliminating over-treatment or low-value care, automating administrative and highly repetitive processes, and allowing the physician to focus on actual care [59,92,93].

Autonomous machines can augment medical professionals, improving the quality of care [4]. In the art of medicine of the future, it is essential to know when to use AI and when we should let natural intelligence work undisturbed [94]. AI and humans form a bio-techno-social system in which each participant contributes something and benefits [95,96]. AI-based systems will support and augment physicians’ skills and are unlikely to replace the traditional physician–patient relationship [4,94,97]. Augmentation means that AI can assist and allow humans to function better. Human clinicians will focus on tasks based on uniquely human skills like big-picture integration, empathy, and persuasion [28,98].

When deep learning’s predictions were combined with a human pathologist, tumor scores increased significantly, and the human error rate decreased [99,100]. AI is expected to become part of the routine radiologists’ work and improve accuracy [101]. Before accessing an actual doctor, AI bots can determine whether specific symptoms warrant a conversation with a physician [102]. Patients are asked multiple questions based on each response, and specific actions are recommended. Medical professionals review these answers for accuracy. Under certain conditions, a referral to a physician with the appropriate level of urgency is provided [103].

AI can also augment patients. Neuroprosthetics help or improve a patient’s nervous system in both input and output via electrical stimulation to overcome neurological deficits [104]. Brain–machine interfaces are platforms where subjects’ intended and voluntary goal-directed wishes are stored and learned when a user trains an AI-based controller and translates them into actions [105].

Nevertheless, these AI systems are not designed for and cannot replace physicians. There will always be cases where a physician’s judgment is needed to define undefined variables or to use variables related to the host, the disease, or the environment that are uncontrollable or hard to quantify or formulate into the algorithm.

A significant challenge in medicine is dealing with uncertainties and decision-making under dynamic circumstances, where the input and output variables are uncontrolled [106]. While this gives the human factor an advantage, AI systems using the Markov Logic Network can handle uncertainty and domain knowledge modeling [28]. Uncertainty modeling is essential for various medical needs, such as monitoring incomplete and unpredictable patients’ activities [28]. These models are necessary for activity recognition, modeling of activities, and decision-making but always require human physician supervision.

## 6. The Constrained Disorder Principle Defines Complex Biological Systems

Variability characterizes biological systems at all levels [107,108,109,110,111]. It is well documented for the genome, cellular functions, and whole organs [112,113,114,115,116,117,118,119,120,121,122,123,124,125,126,127]. Examples are the variability in heart rate, blood pressure, gait, breathing, and brain functions [122,123,124,125,126,127]. The constrained disorder principle (CDP) defines living organisms based on their inherent variability regulated within dynamic borders [5]. Per the CDP, disease states evolve from loss of variability or in cases where the variability is out of control [128].

The CDP implies that variability is mandatory for the normal function of complex systems. It is part of their adaptability and flexibility, which is required for proper function under continuous internal and external perturbations [5,129].

## 7. CDP-Based Second-Generation AI System Augmenting Physicians

CDP-based second-generation AI systems overcome several first-generation systems’ challenges while realizing that using these platforms is to augment and not replace physicians [6]. Second-generation systems are designed to utilize the inherent variability in complex biological processes, better reflecting these processes [112,113,114,115,116,117,118,119,120,121,130]. This co-piloting model of using AI systems by healthcare providers implies augmenting physicians and assisting them in providing better care.

The CDP-based second-generation AI systems implement random patterns into therapeutic regimens and are outcome-driven [6]. Clinical decisions involve numerous complexities that may not have been captured adequately in previous experiences or in the use of big data. Second-generation systems evolve from the *n* = 1 concept, recognizing that each patient is different [6,70,128,131]. It is based on the notion that a subject is never identical to the mean; it is a significant challenge for current big-data-based decision systems [4,132].

AI systems must be adaptive as transformed health ecosystems evolve rapidly and must continuously adapt diagnosis and treatment for each subject. The CDP-based second-generation AI adapts to unforeseen scenarios and provides solutions accounting for biological processes’ dynamicity while personalizing the treatment [6]. AI systems must be context-aware to infer the user’s current activity state and the environment’s characteristics. They also need to be interoperable and to be able to exchange data and knowledge with numerous platforms [8,133].

Second-generation systems are designed to assist decision-making and augment physicians, not replace them. They provide a co-piloting model of an integrated precision medicine solution comprising a patient management tool adapted to the user and connected to data sources. The system offers a method for generating insightful datasets to optimize future diagnostic and therapeutic analysis [6]. See Table 1.

The Digital Pill is a drug regulated by a patent-protected CDP-based second-generation AI system. It is designed to improve drug efficacy by introducing personalized variability into therapeutic regimens [132]. Data-driven variability in dosing and administration times within the approved range is generated. The digital pill is simple for patients and physicians to use and increases product loyalty and adherence, ensuring a long-term sustainable effect [131]. The Digital Pill was suggested to overcome drug resistance and loss of adherence in numerous chronic diseases [121,134,135,136,137,138,139,140,141,142,143,144,145,146,147,148,149,150,151,152,153,154,155,156,157,158,159,160,161,162,163].

Using the system in patients with chronic heart failure and diuretic resistance improved clinical and laboratory parameters, reduced side effects, and minimized heart failure-related emergency room admissions and hospitalizations [153]. The platform stabilized disease progression and improved clinical symptoms in patients with multiple sclerosis. The Digital Pill overcame analgesic tolerance, improving outcomes while reducing side effects in patients with chronic pain [164]. Clinical improvement was shown in patients with the genetic disease Gaucher disease using the system [160]. Additionally, the Digital Pill helped overcome drug resistance in tumors, improving clinical, radiological, and serum biomarkers in patients with cancer [165]. The system was also shown to slow down aging processes [166].

## 8. Adding Value to All Players of the Healthcare System: Reducing Complexity

The CDP-based second-generation AI-based Digital Pill is a companion tool for various healthcare system stakeholders that enables the delivery of precision medicine, streamlines communications, and manages data. These systems generate AI-powered decision-making companions, reducing complexities and adding value for all players of healthcare systems. For physicians and other healthcare providers, it assists decision-making for generating treatment regimens to improve outcomes and adherence. For the patients, these systems improve drug response and reduce side effects. As these systems are outcome-based, they benefit patients where they require the most improved clinical response, ensuring engagement. The systems reduce costs to payers while generating a market disruptor for pharma companies [6,70,131].

The system improves communications with patients and caregivers and provides a data collection and analysis tool to inform better decision-making and improve efficiency and excellence in managing resources. It enables data collection and report performance, supporting service standardization and quality assurance. The Digital Pill can serve as a platform for developing a digital twin for better prediction and risk management and selecting the ideal personalized therapies in a subject-tailored way. It is a tool to manage de-centralized clinical trials, enabling a combination of products with a tool that enhances the benefit of chronic therapies [6,70,131].

The Digital Pill provides solutions to some of the significant challenges in healthcare by overcoming the lack of patient engagement in digital health adoption. It ensures adoption by improving outcomes so patients view the app as part of their treatment. It reduces uncertainty about the opportunities and risks of digital systems. The system allows pharma companies to use existing drugs without the need to develop expensive new products, omits regulatory barriers, reduces costs to the health system, and increases pharma revenues [131].

## 9. Summary

Advances in AI are not just about technology versus humans. AI could be a substitute for many medical activities; however, it is unlikely to reach a stage where it can fully substitute for human physicians. Human doctors will continue serving patients with capabilities augmented by AI. The CDP-based second-generation AI-based Digital Pill is designed to increase physicians’ skills based on a co-piloting model, assisting them in decision-making to improve patients’ responses. The system better reflects biological processes by implementing variability signatures for generating therapeutic regimens. It humanizes AI by moving closer to human biology and using biological systems’ inherent variability to improve outcomes. The system is a physician’s companion, not a replacement for healthcare providers. It restores the efficacy of chronic drugs and improves adherence while generating data-driven therapeutic regimens.

## Figures and Tables

**Table 1:** Potential strengths of second-generation artificial intelligence systems versus first-generation ones.

1. Better reflect biological processes by using the variability inherent to complex routes in biology.
2. Implement random patterns into therapeutic regimens.
3. Outcome-driven.
4. Implement the *n* = 1 concept, recognizing that each patient is different.
5. Dynamically changes as transformed health ecosystems evolve rapidly and must continuously adapt.
6. Adapts to unforeseen scenarios and provides solutions accounting for biological processes’ dynamicity while personalizing the treatment.
7. Provide a co-piloting model of an integrated precision medicine solution.
8. Generate insightful datasets, which optimize future diagnostic and therapeutic analysis.
9. Overcome drug resistance and loss of adherence in chronic disease.
10. Increase patient engagement with the digital system by improving outcomes.
11. Reduce costs to payers by reducing hospitalizations and omitting the need for more expensive medications.
12. Increase revenues to pharma companies.

## Abbreviations

AI: artificial intelligence; ML: machine learning; TS: technological singularity; CDP: constrained-disorder principle; NLP: natural language processing; ANN: artificial neural networks; FES: fuzzy expert systems; EC: evolutionary calculations; HIS: hybrid intelligent systems; DNN: deep neural network; DL: Deep learning.
